# Supplementary material for: Food taboo practices among pregnant women in Deder town, Eastern Ethiopia, 2024
Source: PLoS One. 2025 Apr 16;20(4):e0321773. doi: 10.1371/journal.pone.0321773 (PMC12002467; doi:10.1371/journal.pone.0321773)
Supplement: Attachment — Submitted filename: Food Taboo Manuscript.docx [file pone.0321773.s003.docx]

**Food taboo practices among pregnant women in Deder town, Eastern Ethiopia, 2024**

Abdi Tofik^1^, Tesfaye Gobena^2^, Addis Eyeberu^3^, Adera Debella^3^, Berhe G/Michael^2^, Mulugeta Gamachu^2^, Alemayehu Deressa^2^, Gelana Mamo^2^, Abdi Birhanu^2^, Hamdi Fikradin^2^, Usmael Jibro^3*^_,_ Ibsa Mussa^2^

**Author’s affiliation**

1. Deder general hospital, Eastern Hararge zone, Oromia region, Addis Ababa, Ethiopia.
2. School of Public Health, College of Health and Medical Sciences, Haramaya University, Harar, Ethiopia
3. School of Nursing and Midwifery, College of Health and Medical Sciences, Haramaya University, Harar, Ethiopia

**Authors email address**

AT: [abditofa2004@gmail.com](mailto:abditofa2004@gmail.com) TG: tesfayegob@gmail.com

AE: [addiseyeberu@gmail.com](mailto:addiseyeberu@gmail.com)	AD: aksanadera62@gmail.com

BG: [berhegere09@gmail.com](mailto:berhegere09@gmail.com) MG: [gamachumle@gmail.com](mailto:gamachumle@gmail.com)

ADw: [alexdheressa@gmail.com](mailto:alexdheressa@gmail.com)	GM: gelomamo724@gmail.com

AB: [abdiibiree@gmail.com](mailto:abdiibiree@gmail.com) HF: hamdifekredin@gmail.com

UJ: [usmiye20@gmail.com](mailto:%20usmiye20@gmail.com) IM: [ibsamussa93@gmail.com](mailto:ibsamussa93@gmail.com)

**Correspondence**

- Usmael Jibro: Haramaya University College of Health and Medical Science, Harar, Ethiopia
- O*RCID:* <https://orcid.org/0000-0002-3147-3770>*;*

**Abstract**

**Background**: Nutritional status of mothers during pregnancy and childbirth is affected by maternal food taboo practices. Since the cultural context in eastern Ethiopia is different from others, the study of food taboo practices among pregnant women in eastern Ethiopia is crucial. This could contribute to a better understanding of food taboos practices and inform the development of culturally sensitive interventions to promote optimal nutrition during pregnancy. Therefore, the study aimed to assess the extent of food taboo practices among pregnant women in Deder town, Eastern Ethiopia.

**Method**: An institutional-based cross-sectional study was conducted among 418 pregnant women. The data were collected using a structured interviewer-administered questionnaire. Data were entered into Epi data version 3.02 and then exported to SPSS version 25 for analysis. Binary logistic regression was fitted to identify factors associated with food taboo practices. P-value < 0.05 was used as a cut-off point for statistical significance.

**Results:** The study showed that 56% ((95% CI: 51.2-60.8%) of pregnant women practiced food taboos. Pregnant women who were unable to read and write (AOR: 3.36, 95%CI: 1.24, 9.16), did not have ANC follow-up (AOR=2.04, 95%CI: 1.27, 3.29), food aversion (AOR=2.04, 95%CI: 1.31, 3.18), no additional meal practice (AOR=1.77, 95%CI: 1.14, 2.76), poor knowledge level (AOR=1.96, 95%CI: 1.24, 3.09), and unfavorable attitude (AOR=1.91, 95%CI: 1.22, 2.99) were significantly associated with food taboo practices.

**Conclusion:** More than half of pregnant women practice food taboos. Therefore, nutrition education and awareness creation at health facility service delivery points are necessary actions for pregnant women to avoid food taboo practices.

**Keywords:** Food taboo practice, Pregnant Women, knowledge, attitude, Deder, Eastern Ethiopia

**Introduction**

Food taboos are items that people avoid eating or drinking for religious, cultural, or health reasons (1). Numerous studies from Asia (2-5) and Africa (5-9) have shown that women are compelled by traditional beliefs to forgo healthful diets throughout pregnancy and the postpartum period. The more prevalent taboos, albeit being of varied kinds, were related to the concurrent use of milk and milk products, eggs, linseed, fatty meats, fruits including mango, orange, pineapple, nuts, and vegetables (10-13). Evidence showed that these food taboos contribute to the undernutrition of mothers and fetuses during pregnancy, which in turn harms their health [13]. In contrast to this, research done on the adaptive explanation of food taboo practice in Fiji showed that food taboos reduce women's chance of fish poisoning by 30% during pregnancy(14). Another study done in India showed that pregnant women avoided food such as fruits to protect their fetuses which is acquired through learning(15)

In developing nations, it is customary to limit one's food intake when pregnant. There have been reports of widespread food taboo practices around the world. The prevalence of food taboos among pregnant women in Malaysia was 70.2% (16), in South Africa was 37% (13), and in Nigeria was 66% (17). In Ethiopia, the prevalence of food taboos ranged from 11.5% to 55.3% (7, 9, 10, 18). However, according to a systematic review and meta-analysis study, the prevalence of food taboo practices was reported high in Ethiopia at 34.22% (11). Regarding pregnant women's perception of food taboos, many women in Ethiopia have traditional beliefs relating to pregnancy and misconceptions about weight gain during pregnancy (7, 19)

The avoidance of certain foods during pregnancy is thought to be influenced by a variety of variables, including culture, lack of nutritional counseling during antenatal care (ANC) visits, younger age, lower educational attainment, poor income, being multiparous, living in a rural area, and disliking of particular foods (9, 11, 20, 21). Nevertheless, a sizable portion of pregnant women refrain from eating particular foods because of cultural traditions or expectations. The concerns mentioned include worries about having a big baby, unpleasant vaginal discharge during birth, and skin conditions on the body (22). Evidence shows that most women learn food taboos from their families and transmit them to society. Due to this women have strong beliefs in their culture (7, 13). Evidence recommended that culturally appropriate interventions and indigenous knowledge about the food suggestions could be a good solution to enhance maternal nutrition (13, 23)

The practice of food taboos has a significant negative influence on the health of new mothers and their babies. Dietary deficiencies, which have detrimental effects on both the mother and the newborn, are the most frequent consequence of food taboo (24). The risk of premature birth, LBW, and less optimum growth and development of children may increase due to the mother's poor nutritional status during pregnancy (20, 23, 25). Maternal death, psychological distress caused by poor fetal outcomes, and anemia are further serious effects of dietary inadequacy for women (26). Food taboos might also be adaptive and not always harmful to health (14, 15, 27).

Ethiopia is attempting to enhance the nutritional status of pregnant women by implementing policies and initiatives from international health recommendations. According to numerous publications, nutritional counseling during ANC, micronutrient supplements, iron, and folic acid supplementation, and other interventions have a positive impact on women's nutritional status (28-33).

Food avoidance during pregnancy is still an unresolved health concern in Ethiopia, even though the nutritional status and eating behaviors of Ethiopian women during pregnancy have been assessed (34, 35). Furthermore, the cultural context in eastern Ethiopia is different from other regions of the country, so the study of food taboo practices among pregnant women in eastern Ethiopia is crucial. This could contribute to a better understanding of the effect of food taboos on maternal and fetal health outcomes in Ethiopia and inform the development of culturally sensitive interventions to promote optimal nutrition during pregnancy. By analyzing the gap surrounding food taboos, it is crucial to preserve the good nutritional state of pregnant women. Therefore, this study aimed to assess the food taboos among pregnant women in eastern Ethiopia.

**Materials and Methods**

**Study design, area, and period**

During the first to the last week of February 2022, a cross-sectional study was carried out in Deder Town's public health facilities in eastern Ethiopia. Deder, a town in Oromia, Ethiopia, is situated in the East Hararge Zone at a height of 2,117 meters (6,946 feet) above sea level. It is situated 458 kilometers east of Addis Ababa, the capital of Ethiopia. According to the population forecast for 2030, the town has a total anticipated population of 32,656, of which 16,987 were men and 15,669 were women. The community is home to 10 private clinics, five health posts, one general hospital, and one health center. More than 1.5 million patients from six districts are referred to Deder General Hospital.

**Populations and Criteria**

Pregnant women who attended ANC follow-ups in public health facilities (Deder General Hospital and health centers) were the study population. All pregnant women who attended ANC at public health facilities were included in the study. Pregnant women who were unable to respond during the data collection period were excluded.

**Sample Size Determination and Sampling Procedure**

The minimum required sample size for the study was determined by using a single population proportion formula under the following statistical assumption: P= 55.3% (2), =5%, a 95% CI. Then by considering a 10% non-response rate, the final sample size became 418.

Two public healthcare facilities that offer ANC services (Deder Hospital and Deder Town Health Center) were chosen for the studyt. The determined sample size, n=418, was proportionally distributed taking into account the monthly patient flow rate of pregnant women for ANC utilization in the health institutions. As a result, the current study's determined sample size (418) included a total of 247 participants from Deder General Hospital and 170 participants from Deder Town Health Center. Additionally, a method of systematic random sampling was used to sample our study participants using pregnant women's identification numbers. The first sample was selected randomly between the first ANC attendee's mother and the 2nd attendee.

**Data Collection procedure and quality control**

The structured questionnaire was adapted from previous similar studies (7, 12, 36, 37). The tool had five parts which included sociodemographic characteristics such as age, residency, ethnicity, religion, educational status, occupational status, and monthly income; obstetrics-related characteristics such as parity, ANC follow-up, gestational age, and source of nutritional counseling; dietary pattern related items such as mean eating frequency, additional meal practice, and skipping meal practice; food aversion and craving related items (both open-ended and close-ended items) such as food aversion, type of food averted, reason for aversion, food craving, and reason for food craving; knowledge related questions; and attitude related question(both open-ended and close-ended items). The questionnaires were prepared in English and then translated into the local languages of the study area (Afan Oromo and Amharic). The questions were then translated back into English to ensure consistency and correctness of the translation. Any discrepancies in meaning between the original and the back-translated version are then fixed. The questionnaire was pre-tested on 5% of the sample size in the nearby Woreda health center before the data collection, and adjustments were made depending on the results. Subsequently, the instrument's validity and reliability were examined. We measured the instruments' quality using Cronbach's Alpha, which was 0.83. Seven different health professions gathered the data. Data collection started on February 01, 2022, and ended on February 30, 2022. For data collectors and supervisors, a two-day training on the study's goal and data gathering instrument was provided. The supervisors and investigator double-checked the surveys every day. Each completed questionnaire was reviewed for consistency and completeness.

**Measurements**

Pregnant women's attitudes regarding food taboo practices during pregnancy were assessed using seven 5-point Likert scale attitude questions. All of the questions were rated on a scale of 1 to 5 (1 being strongly disagreed, 2 disagreed, 3 neutral, 4 agreeing, and 5 strongly agreeing). The mean score was used as a cut point after the normalcy was checked, and participants with a mean score of 50% or higher were deemed to have positive attitudes toward food taboo practices, while those with a score below 50% were deemed to have negative attitudes (36, 38). To evaluate women's level of knowledge on food taboos during pregnancy, ten knowledge assessment questions were used. Each correct response received a score of 1, while incorrect answers received a score of 0. Following a normalcy check, a cut point of the mean score was determined. Participants were classified as having good knowledge if their mean score was 50% or higher, and as having poor knowledge if their mean score was less than 50% (36, 38).

Additional meal practices: The Essential Nutritional Action (ENA 2015) recommends pregnant

women eat at least one extra meal per day (39).

Frequency of regular meals: The Institute of Medicine recommends that pregnant women eat regular meals at least three times a day (40).

Food taboo: Food taboo is the deliberate avoidance of at least one food item for religious, cultural, and social reasons other than a simple dislike of food preference.

**Data Processing and Analysis**

Epidata 3.02 was used to enter the data, which was then exported to SPSS 25.0 for additional statistical analysis. The characteristics of the study participants were described using means, medians, frequencies, and percentages. The significant variables associated with the outcome variables were found using binary logistic regression analysis. Before fitting the model, the variance inflation factor (VIF) and tolerance test were used to evaluate the multicollinearity assumption for each covariate. All independent variables were fitted with the bivariate model, and variables with p-values less than 0.25 were included in the multivariate logistic regression analysis. To control the confounding variables and identify the related factors, multivariable logistic was fitted last. With a 95% confidence interval, the adjusted odds ratio was used to determine the strength of the association. At a p-value of 0.05, statistical significance was declared. Hosmer and Lemeshow tests were used to evaluate the model's goodness of fit.

**Ethical Consideration**

The Institutional Health Research Ethics Review Committee (IHRERC) of Haramaya University's College of Health and Medical Sciences granted its approval with the reference number IHRERC/230/202. The study sought permission to conduct the research from public health institutions of Deder town. After the study's purpose, data collection method, benefits, and risks were fully disclosed to the study participants, they gave their fully informed, voluntary, written, and signed consent, and the data were collected. *For participants under 18 years of age, written informed consent was obtained from their parent/guardian.*

**RESULTS**

**Socio-demographic Characteristics**

A total of 418 pregnant women participated in this study, resulting in a response rate of 98%. The mean (+SD) age of the study participant was 25.8 (±3.901) years. Of the total study participants, 409(97.8%), 175(41.9%), and 198(47.4%) women who were married attended primary school and had a family size of 1-3 respectively **(Table 1).**

**Obstetrics related factors**

More than half 212(50.7%) of pregnant women had previous Antenatal care follow-ups. Regarding sources of nutrition information: more than half 216(51.7%) of the respondents got their nutrition information from their close relatives, followed by 116(27.8%) friends **(Table 2).**

**Knowledge and Attitude of Pregnant Women about Food Taboo Practices**

Food taboos were familiar to most of the study participants as 342 (81.8%) respondents had heard of food taboos. More than half 225 (53.8%) of the study participants had poor knowledge scores. The mean knowledge score was 4.64±2.129 SD with a minimum score of 2 and a maximum score of 10. Regarding attitudes, 226 (54.1%) of the respondents had unfavorable attitudes toward food taboos practice. The majority of pregnant women 279(66.7%) did not believe that vegetables cause unpleasant smells in newborns and mothers, while 272(65.1%) of pregnant women believed that honey causes abortion **(Table 3).**

**Eating Habits of Pregnant Women**

Of the 418 respondents, 226 (54.1%) and 240 (57.4%) never consumed fruits and vegetables respectively during the current pregnancy, while 117 (61%) and 92 (52%) consumed fruits and vegetables once per day, respectively. Regarding meal patterns; the majority 215(51.4%) of the respondents did not eat an additional meal during the current pregnancy and the main reason for not taking additional meals was the fear of a big baby 108(50.2%) followed by the fear of weight gain 89(41.4%). Regarding the practice of skipping meals, 133(31.8%%) of respondents skipped at least one meal per day **(Table 4).**

Among the 418 respondents, 211 (50.5%) had consumed coffee during the current pregnancy 155 (73.5%) had drunk less than two cups of coffee per coffee ceremony while only 56 (26.5%) drank more than two cups of coffee per coffee ceremony.

**Food Aversions and Craving among Pregnant Women**

Out of 418 respondents, 218 (52.2%) developed food aversion during the current pregnancy. The food avertions were; 89(40.8%) averted cereal products, followed by eggs 77 (35.3%)**.** The main reasons for food aversion were 121 (55.5%) due to nausea and vomiting, followed by 77 (35.3%) smell of foods**.** The trimester food aversion developed was 114 (52.3%) first trimester followed by, 61 (28%) third trimester (**Table 5).**

Regarding pica. among the 418 respondents, 319 (76.3%) respondents did not develop cravings for unnatural substances while only 99 (23.7%) respondents experienced the consumption of pica. Among those who experienced the consumption of pica, 78 (78.8%) consumed clay followed by 14 (14.1%) and 7 (7.1%), consumed ash and ice respectively.

**The Magnitude of Food Taboo Practices among Pregnant Women**

Two hundred thirty-four (56%) of the respondents avoided at least one food item during the current pregnancy for various traditional beliefs and cultures with a 95% CI of 51.2-60.8%. Among those who practiced food taboos, 81 (34.6%) considered fruits as taboos, followed by 43 (18.4%) vegetables, 38 (18.4%) honey, 36(15.4%) milk products, 28 (12%) meat, and 8 (3.4%) egg**.** Regarding reasons for avoiding these foods; A common belief underpinning many of the taboos was difficulty during childbirth because the baby will be big 109 (46.6%), followed by 44 (18.8%) abdominal cramps for the mother and fetus, the unnecessary thing will be coated on fetal body 43 (18.4%), and causes miscarriage (15.4%).

**Factors Associated with Food Taboo Practices among Pregnant Women**

In the multivariate analysis, pregnant women's educational status (unable to read and write), partner’s educational status (primary education), no previous ANC visit, having food aversion, no additional meal practice, poor knowledge of food taboos, and unfavorable attitude toward food taboos were significantly associated with food taboo practice.

Pregnant women who were unable to read and write had three times higher odds of practicing food taboo practices (AOR=3.36, 95% CI: 1.24, 9.16) as compared to those who had attended college and above. Pregnant women who had no previous ANC follow-up had two times higher odds of practicing food taboos (AOR=2.04, 95% CI: 1.27, 3.3) compared to their counterparts.

Pregnant women with food aversion had two times higher odds of practicing food taboos (AOR=2.04, 95% CI:1.308, 3.18) compared to their counterparts. The odds of practicing food taboos were nearly two times higher among pregnant women without additional meal practices (AOR=1.8, 95% CI: 1.14, 2.76), having poor knowledge of food taboos (AOR=1.96, 95% CI: 1.24, 3.09), having unfavorable attitudes (AOR=1.91, 95% CI: 1.22, 2.99) compared to their counterparts respectively (**Table 6**).

**Discussion**

This study measured the extent of practices surrounding food taboos. Furthermore, it was discovered that food taboo practices were substantially correlated with pregnant women's educational status, their husbands' educational status, their ANC follow-up, their food aversion, their supplementary meal practice, their nutritional understanding, and their attitude toward nutrition.

We found that the prevalence of food taboo practices was 56% (95% confidence interval: 51.2-60.8%). This result is consistent with research conducted in the Ethiopian towns of Shashamane (49.8%)(7) Mandura (55.2%) (41), and Sendafa Bake (55.3%) (10). This finding is higher than studies conducted in Ethiopia Mekele City (11.5%) (18) and Awebel District (27%) (9). The possible justification could be variations in sociodemographic characteristics, research site, ANC service coverage, sample size, and sampling techniques. The results of this study were also lower than those of studies from Malay 70.2% (16) and Papua New Guinea 66% (42). This may be due to variations in cross-cultural differences, economic status, study location, and inclusion standards. Another explanation for the high prevalence of food taboos is that women may assume food taboos can help preserve cultural traditions and practices, fostering a sense of identity and community (27). Additionally, in some cases, food taboos may discourage the consumption of foods that are more prone to contamination or spoilage, thus reducing the risk of foodborne illnesses some food taboos may have health benefits such as avoiding certain foods that are known allergens or that may exacerbate certain medical conditions in susceptible individuals(14). All those conditions may lead to the high prevalence of food taboos practice.

This finding implied that food taboos are widely practiced among pregnant women across the globe. A meta-analysis study showed that pregnant women avoid certain foods due to cultural concerns about birth weight and clinical evidence of obstructed labor in Africa, Asia, and Europe (43). This study finding illustrated that policymakers must prioritize education and awareness campaigns to address prevalent food taboos among pregnant women. Empowering them with accurate nutritional information ensures maternal and fetal health is not compromised.

We found a strong association between education level and food taboo practices. This finding is consistent with research from Sudan, Shashamane, Mekele, and Sendafa Bake in Ethiopia. (7, 10, 18, 44). The conceivable explanation could be that women have no formal education are unable to acquire knowledge concerning the effects of food taboos and take into account cultural myths that promote avoiding food. Additionally, individuals could follow food taboos because they lack knowledge from formal education and are unable to study useful literature.

Pregnant women whose husbands had attended primary school were more likely to observe food taboos than their counterparts. This finding is consistent with a study conducted in Sidama Ethiopia (45). This may be because husbands with less education are unable to provide their wives with nutritional advice due to a lack of expertise. This result suggested that there is a glaring informational gap in the effects of dietary abstinence during pregnancy. Therefore, it is preferable to work on raising awareness for both women and their families.

In this study, not having ANC follow-up was significantly associated with food taboo practices. Pregnant women who have never had ANC follow-up were more likely to practice food taboos as compared with pregnant women who have had ANC follow-up. This finding is in harmony with studies conducted in Mandura and Awebel districts in Ethiopia (41, 46). The reason could be that mothers who never had ANC follow-up will miss out on nutritional counseling services and awareness-raising activities given by healthcare professionals, which will ultimately raise food taboo practices. The study's findings, which show that relatives like the mother, grandmother, mother-in-law, and friends are the pregnant women’s primary sources of dietary information rather than medical facilities or other professionals, support this. This result suggested that improving ANC follow-up will enhance or improve their comprehension of the effects of food taboos.

We found a strong correlation between food aversion and food taboo practices. Studies conducted in southwest India, Sendafa Bake town, Ethiopia, and Sidama Zone, Ethiopia, confirm this conclusion (10, 27, 45). A change in olfactory and taste sensitivity, which could result in nausea, might be considered a possible factor contributing to the development of food aversion in pregnant women. It was also found that additional meal practice was also significantly associated with a pregnant woman's practice of food taboos.

Pregnant women who did not eat an extra meal during pregnancy were more likely to have food taboos than their counterparts. This finding is similar to studies conducted in Khartoum, Sudan, Sendafa Bake town, and Mandura Woreda, Ethiopia. This might be due to wrong beliefs or religious and cultural views. As identified in all studies pregnant women avoided the extra meal due to fear of gaining weight, fear of difficulty in childbirth, and lack of appetite (10, 41, 44).

Furthermore, the study also identified that pregnant women's attitudes were also significantly associated with food taboos. Accordingly, 54.1% of pregnant women have an unfavorable attitude toward food taboos which is in agreement with the study conducted in the Shashamane District, Ethiopia (49.8%) (7), and Pondicherry, South India (63.7%) (24). This similarity might be due to adherence to the religious and cultural practices, educational status, and residence of study participants.

**Strengths and limitations of the study**

The strength of the study is that it assessed pregnant women's knowledge and attitudes toward food taboo practices that had not been previously evaluated by other researchers. However, since it was only a quantitative method it cannot provide an in-depth explanation of why pregnant women practice food taboos. Also, because it was a facility-based study, the results of the study cannot be generalized to all pregnant women in the study area. Furthermore, this study used both male and female interviewers and the study may be influenced by interviewer effects as women may not discuss all practices with male interviewers.

**Conclusion**

More than half of the pregnant women in this study were restricted from at least one food item because they believed in traditional and cultural beliefs. Pregnant women unable to read and write, pregnant women whose husbands attended primary school, had no previous ANC follow-up, had food aversion, had no additional meal practice, had poor knowledge of food taboos, and unfavorable attitudes toward food taboos were more likely to practice food taboos. The food items that were avoided during pregnancy were fruits, vegetables, honey, milk and dairy products, fatty meat, and eggs. The reasons for avoiding these food items were fear of difficulties during childbirth due to the increased size of the fetus, causing abdominal cramps to mother and fetus, attaching to the fetal body and causing an offensive odor to the mother and newborn baby, and fear of miscarriage. It's important to note that while there may be perceived advantages to food taboos, they should be considered within the broader context of cultural, nutritional, and health considerations. Women who had restricted access to essential nutrients led to dietary imbalances. Cultural appropriate interventions and nutritional counseling during ANC visits could be a good solution to enhance maternal nutrition.

**Declarations**

**Ethics approval and consent to participate**

The Institutional Health Research Ethics Review Committee (IHRERC) of Haramaya University's College of Health and Medical Sciences granted its approval with the reference number IHRERC/230/202. Written, and signed consent was obtained from all participants and confidentiality was assured. For participants under 18 years of age, written informed consent was obtained from their parent/guardian. This research was done following Hilinski Declarations and national ethical guidelines.

**Consent for publication:** Not applicable

**Availability of data and materials:**

In this study, all pertinent information is given. However, the corresponding author will provide more information upon reasonable request.

**Competing Interests.**

The authors declare that there is no competing interest in this work.

**Author contributions**

All authors participated in the development of the manuscript as well as the data review, drafting, methodology, data analysis, and drafting of the results. The final draft of the manuscript to be published has been read and approved by all authors, who also concur on all other aspects of this work.

**Acknowledgments**

We would like to express our sincere gratitude to Haramaya University for supporting us in writing this work, as well as to the data collectors and supervisors.

**References**

1.	Maduforo A, Nwosu O, Ndiokwelu C, Obiakor-Okeke P. Food superstition, feeding practices and nutritional anthropometry of pregnant women. Jorind. 2013;11:1.

2.	Patil R, Mittal A, Vedapriya D, Khan M, Raghavia M. Taboos and misconceptions about food during pregnancy among rural population of Pondicherry. Calicut Med J, 2010. 8 (2), 4.

3.	Banu KK, Prathipa A, Anandarajan B, Sheriff AMI, Muthukumar S, Selvakumar J. Food taboos during antenatal and postpartum period among the women of rural and urban areas of Tamilnadu. Int J Biomed. 2016;7(8):393-6.

4.	Shwetha T, Shwetha R, Krishna I, Usha R. Food taboos among pregnant and lactating mothers in Tumkur: a qualitative study. Int J Commun Med Public Health. 2017;4(4):1060-5.

5.	Otoo P, Habib H, Ankomah A. Food prohibitions and other traditional practices in pregnancy: a qualitative study in western region of Ghana. Advances in Reproductive Sciences. 2015;3(03):41.

6.	Desalegn K, Pragya S, Debebe M. .Dietary Practices and Associated factors among Pregnant Women in Wondo Genet District, Southern Ethiopia: A cross-sectional study. Journal of Pharmaceutical and Scientific Innovation. 2015.

7.	Zepro NB. Food taboos and misconceptions among pregnant women of Shashemene District, Ethiopia, 2012. Sci J Public Health. 2015;3(3):410-6.

8.	Oluleke M, Ogunwale A, Arulogun O, Adelekan A. Dietary intake knowledge and reasons for food restriction during pregnancy among pregnant women attending primary health care centers in Ile-Ife, Nigeria. Int J Popul Stud. 2016;2(1):103-16.

9.	Getnet W, Aycheh W, Tessema T. Determinants of food taboos in the pregnant women of the awabel district, east gojjam zone, amhara regional state in Ethiopia. Advances in Public Health. 2018;2018.

10.	Wondimu R, Tesfahun E, Kaba Z. Food Taboo and Its Associated Factors Among Pregnant Women in SendafaBeke Town, Oromia Regional State, Ethiopia. International Journal of Science, Technology and Society. 2021;9(2):75.

11.	Debela BG, Sisay D, Hareru HE, Ewune HA, Tesfa A, Shewaye DA, et al. Food taboo practices and associated factors among pregnant women in Ethiopia: a systematic review and meta-analysis. Scientific Reports. 2023;13(1):4376.

12.	Mohammed SH, Taye H, Larijani B, Esmaillzadeh A. Food taboo among pregnant Ethiopian women: magnitude, drivers, and association with anemia. Nutrition journal. 2019;18:1-9.

13.	Chakona G, Shackleton C. Food taboos and cultural beliefs influence food choice and dietary preferences among pregnant women in the Eastern Cape, South Africa. Nutrients. 2019;11(11):2668.

14.	Henrich J, Henrich N. The evolution of cultural adaptations: Fijian food taboos protect against dangerous marine toxins. Proceedings of the Royal Society B: Biological Sciences. 2010;277(1701):3715-24.

15.	Placek CD, Hagen EH. Fetal protection: the roles of social learning and innate food aversions in South India. Human Nature. 2015;26:255-76.

16.	Mohamad M, Ling CY. Food taboos of Malay pregnant women attending antenatal check-up at the maternal health clinic in Kuala Lumpur. Integrative Food, Nutrition and Metabolism. 2016;3(1):262-7.

17.	Olarinoye A, Adesina K, Olarinoye J, Adejumo A, Ezeoke G. Food taboos among pregnant Nigerian women. 2014.

18.	Tela FG, Gebremariam LW, Beyene SA. Food taboos and related misperceptions during pregnancy in Mekelle city, Tigray, Northern Ethiopia. PLoS One. 2020;15(10):e0239451.

19.	Zerfu TA, Umeta M, Baye K. Dietary habits, food taboos, and perceptions towards weight gain during pregnancy in Arsi, rural central Ethiopia: a qualitative cross-sectional study. Journal of Health, Population and Nutrition. 2016;35(1):1-7.

20.	Teshome A, Zinab B, Wakjira T, Tamiru D. Factors associated with food taboos among pregnant women in the Dimma district, Gambella, Ethiopia. African Journal of Midwifery and Women's Health. 2020;14(4):1-9.

21.	Demissie T, Muroki N, Kogi-Makau W. Food taboos among pregnant women in Hadiya Zone, Ethiopia. Ethiopian Journal of Health Development. 1998;12(1).

22.	Assefa D, Wassie E, Getahun M, Berhaneselassie M, Melaku A. Harmful traditional practices for the Ethiopian health center team. Hawassa, College. 2005:33-40.

23.	Diana R, Rachmayanti RD, Anwar F, Khomsan A, Christianti DF, Kusuma R. Food taboos and suggestions among Madurese pregnant women: a qualitative study. Journal of Ethnic Foods. 2018;5(4):246-53.

24.	Rajkumar P, Anuj M, Vedapriya D, Khan MI, Raghavia M. Taboos and misconceptions about food during pregnancy among rural population of Pondicherry. Calicut Medical Journal. 2010;8(2).

25.	Ekwochi U, Osuorah CD, Ndu IK, Ifediora C, Asinobi IN, Eke CB. Food taboos and myths in South Eastern Nigeria: The belief and practice of mothers in the region. Journal of ethnobiology and ethnomedicine. 2016;12(1):1-6.

26.	Khanna SB, Dash K, Swasti DK. Fetal origin of adult disease. JK Sci. 2007;9(4):206-10.

27.	Placek CD, Madhivanan P, Hagen EH. Innate food aversions and culturally transmitted food taboos in pregnant women in rural southwest India: Separate systems to protect the fetus? Evolution and Human Behavior. 2017;38(6):714-28.

28.	Demilew YM, Alene GD, Belachew T. Effect of guided counseling on nutritional status of pregnant women in West Gojjam zone, Ethiopia: a cluster-randomized controlled trial. Nutrition journal. 2020;19(1):1-12.

29.	Ghosh S, Spielman K, Kershaw M, Ayele K, Kidane Y, Zillmer K, et al. Nutrition-specific and nutrition-sensitive factors associated with mid-upper arm circumference as a measure of nutritional status in pregnant Ethiopian women: implications for programming in the first 1000 days. PloS one. 2019;14(3):e0214358.

30.	Kaleem R, Adnan M, Nasir M, Rahat T. Effects of antenatal nutrition counselling on dietary practices and nutritional status of pregnant women: A quasi-experimental hospital based study. Pakistan Journal of Medical Sciences. 2020;36(4):632.

31.	Organization WH. Essential nutrition actions: improving maternal, newborn, infant and young child health and nutrition. 2013.

32.	Organization WH. Global nutrition targets 2025: low birth weight policy brief. World Health Organization; 2014.

33.	Organization WH. Guideline: daily iron and folic acid supplementation in pregnant women: World Health Organization; 2012.

34.	Vasilevski V, Carolan-Olah M. Food taboos and nutrition-related pregnancy concerns among Ethiopian women. J Clin Nurs. 2016;25(19-20):3069-75.

35.	Diddana TZ. Factors associated with dietary practice and nutritional status of pregnant women in Dessie town, northeastern Ethiopia: a community-based cross-sectional study. BMC Pregnancy and Childbirth. 2019;19(1):517.

36.	Mengie T, Dessie Y, Egata G, Muche T, Habtegiorgis SD, Getacher L. Food taboos and associated factors among agro-pastoralist pregnant women: A community-based cross-sectional study in Eastern Ethiopia. Heliyon. 2022;8(10).

37.	Amare W, Tura AK, Semahegn A, Teji Roba K. Food taboos among pregnant women and associated factors in eastern Ethiopia: A community-based cross-sectional study. SAGE open medicine. 2022;10:20503121221133935.

38.	Payghan B, Kadam S, Reddy R. A comparative study of nutritional awareness among urban-rural pregnant mothers. Res Rev J Med Health Sci. 2014;3:95-9.

39.	Guyon A, Quinn V, Nielsen J, Stone-Jimenez M. Essential Nutrition Actions and Essential Hygiene Actions Training Guide: Community Workers. Washington, DC: Core Group. 2015.

40.	Maria Siega-Riz A, Herrmann TS, Savitz DA, Thorp JM. Frequency of Eating During Pregnancy and Its Effect on Preterm Delivery. American Journal of Epidemiology. 2001;153(7):647-52.

41.	Alga A. FOOD TABOO AND ASSOCIATED FACTORS AMONG PREGNANT WOMEN ATTANDING ANC SERVICE IN MANDURA WOREDA, BENISHANGUL GUMUZ REGIONAL STATE, NORTHWEST ETHIOPIA 2020.

42.	Kuzma J, Paofa D, Kaugla N, Catherina T, Samiak S, Kumei E. Food taboos and traditional customs among pregnant women in Papua New Guinea: Missed opportunity for education in antenatal clinics. Contemporary PNG Studies. 2013;19:1-11.

43.	Maggiulli O, Rufo F, Johns SE, Wells JC. Food taboos during pregnancy: meta-analysis on cross cultural differences suggests specific, diet-related pressures on childbirth among agriculturalists. PeerJ. 2022;10:e13633.

44.	Tahir HMH, Mohammed AEE, Mohammed NAA. Food Taboos among Pregnant Women in Health Centers, Khartoum State- Sudan,. Austin Journal of Public Health and Epidemiology. 2016;5(2): .

45.	Handiso YH. Prevalence of food aversions, cravings and pica during pregnancy and their association with nutritional status of pregnant women in Dale Woreda, Sidama zone, SNNPRS, Ethiopia. International Journal of Nutrition and Metabolism. 2015;7(1):1-14.

46.	Wollelaw Getnet, Wubie Aycheh, Tessema T. Determinants of food taboos in the pregnant women of the awabel district, east gojjam zone, amhara regional state in Ethiopia. Advances in Public Health. 2018;2018:6.

**List of tables**

Table 1: Socio-demographic characteristics of pregnant women attending ANC follow-up in Deder town health facilities, Eastern Ethiopia, 2022(N=418).

| **Variables** | **Category** | **Frequency** | **Percent** |
| --- | --- | --- | --- |
| Age groups | <=19 | 18 | 4.3 |
|  | 20-24 | 144 | 34.4 |
|  | 25-29 | 162 | 38.8 |
|  | 30-34 | 91 | 21.8 |
|  | >=35 | 3 | 0.7 |
| Ethnicity | Oromo | 346 | 82.8 |
|  | Amhara | 29 | 6.9 |
|  | Gurage | 23 | 5.5 |
|  | Harari | 10 | 2.4 |
|  | Other* | 10 | 2.4 |
| Religions of mother | Muslim | 324 | 77.5 |
|  | Orthodox | 43 | 10.3 |
|  | Protestant | 38 | 9.1 |
|  | Waqefata | 13 | 3.1 |
| Current marital status | Married (live together) | 409 | 97.8 |
|  | Divorced | 4 | 1.0 |
|  | Widowed | 5 | 1.2 |
| Mother educational status | Unable to read and write  Primary school  Secondary school  College and above | 68  175  113  62 | 16.3  41.9  27.0  14.8 |
| Occupations of mother | Housewife  Gov't employee  Merchant  Other** | 268  55  88  7 | 64.1  13.2  21.1  1.7 |
| Husband educational level | Unable to read and write  Primary school  Secondary school  College and above | 87  77  144  110 | 20.8  18.4  34.4  26.3 |
| Average monthly income | <=1000  1001-2000  2001-3000  3001-4000  >=4000 | 177  21  120  16  84 | 42.3  5.0  28.7  3.8  20.1 |
| Residence of mother | Rural  Urban | 293  125 | 70.1  29.9 |

***** Somali, Silte**. **** Student	******* driver, daily laborer**,**

Table 2: Obstetrics-related factors of pregnant women attending ANC follow-up in Deder town health facilities, Eastern Ethiopia, 2022(N=418).

| **Variables** | **Category** | **Frequency** | **Percent** |
| --- | --- | --- | --- |
| Family size | 1-3  4-6  >=7 | 198  169  51 | 47.4  40.4  12.2 |
| Number of birth(parity) | ≤ 2  3-6  ≥ 7 | 214  171  33 | 51.2  40.9  7.9 |
| Previous ANC follow-up | Yes  No | 212  206 | 50.7  49.3 |
| GA at the first ANC visit | <=12wks  12-27wks  >=28wks | 49  221  148 | 11.7  52.9  35.4 |
| Sources of nutrition information | Close relatives  Friends  Health workers  Others***** | 216  116  37  49 | 51.7  27.8  8.9  11.7 |

***** TV, radio, newspaper, social media

Table 3: Knowledge, and attitudes of pregnant women attending ANC follow-up in Deder town health facilities, Eastern Ethiopia, 2022(N=418).

| **Knowledge questions** | | **Yes N(%)** | | **No N (%)** | |
| --- | --- | --- | --- | --- | --- |
| Have you heard about the taboo for pregnant women during pregnancy? | | 342(81.8) | | 76(18.2)* | |
| Fatty/oily foods are taboo for pregnant women | | 324(77.5) | | 94(22.5)* | |
| Milk and its products are taboo for pregnant women | | 313(74.9) | | 105(25.1)* | |
| Bananas are taboo for pregnant women | | 331(79.2) | | 87(20.8)* | |
| An egg is a taboo for pregnant women | | 307(73.4) | | 111(26.6)* | |
| Potato is taboo for pregnant women | | 22(5.3) | | 396(94.7)* | |
| Cabbage is taboo for pregnant women | | 210(50.2) | | 208(49.8)* | |
| Honey is taboo for pregnant women | | 221(52.9) | | 197(47.1)* | |
| Sugarcane is taboo for pregnant women | | 94(22.5) | | 324(77.5)* | |
| Pumpkin is taboo for pregnant women | | 78(18.7) | | 340(81.3)* | |
| **Knowledge status Good** (mean score ≥5) =193(46.2%)  **Poor** (mean score <5) =225(53.8%) | | | | | |
| **Attitude related questions** | **Agree**  **N(%)** | | **Unsure**  **N(%)** | | **Disagree**  **N(%)** |
| Vegetables coated on the fetal body cause newly born babies and mothers to bad smell | 135(32.3) | | 4(1) | | 279(66.7)* |
| Fatty meat and butter coated on the fetal body cause the fetus to rise down in the womb and make difficulties during delivery | 212(50.7) | | 0 | | 206(49.3)* |
| Read meat causes the baby too big in the womb and causes prolonged labor | 209(50) | | 0 | | 209(50)* |
| Fruits cause the baby too big in the womb and cause difficulties during delivery | 214(51.2) | | 1(0.2) | | 203(48.6)* |
| Honey cause abortion | 272(65.1) | | 1(0.2) | | 145(34.7)* |
| Mustered cause abortion | 135(32.3) | | 4(1) | | 279(66.7)* |
| spicy food causes the baby’s hair to thin | 209(50) | | 0 | | 209(50)* |
| **Attitude status Favorable** (mean score>10) =192(45.9%)  **Unfavorable (**mean score ≤10) = 226(54.1%) | | | | | |

* Correct answer

Table 4: Meal patterns of pregnant women attending ANC follow-up in Deder town health facilities, Eastern Ethiopia, 2022(N=418).

| **Variables** | **Category** | **Frequency** | **Percentage** |
| --- | --- | --- | --- |
| Regular meal eating frequency | Once  Twice  Three times  ≥ Four times | 37  116  216  49 | 8.9  27.8  51.7  11.7 |
| Additional meal practices | Yes  No | 203  215 | 48.6  51.4 |
| Number of additional meal practice | One  Two  Three | 127  58  18 | 62.6  28.6  8.9 |
| Reasons for not taking additional meal | Fear big baby  Fear of weight gain  Lack of appetite | 108  89  18 | 50.2  41.4  8.4 |
| Skipping meal practice | Yes  No | 133  285 | 31.8  68.2 |
| Types of meal skipped | Breakfast  Lunch  Dinner | 77  26  30 | 57.9  19.5  22.6 |
| Reasons for skipping | Fear of nausea and vomiting  Abdominal discomfort  Heartburn  Fasting | 38  14  68  13 | 28.6  10.5  51.1  9.8 |

Table 5: Food aversions and cravings among pregnant women attending ANC follow-up in Deder town health facilities, Eastern Ethiopia, 2022(N=418).

| **Variables** | **Category** | **Frequency** | **Percent (%)** |
| --- | --- | --- | --- |
| Food aversion | Yes  No | 218  200 | 52.2  47.8 |
| Types of food averted | Cereal products (Pasta, Rice, and Spaghetti)  Egg  Fatty meat  Spicy foods | 89  77  32  20 | 40.8  35.3  14.7  9.2 |
| Reasons for food aversion | Nausea and vomiting  Smell of food  Heartburn | 121  77  20 | 55.5  35.3  9.2 |
| Trimester aversion  Developed | 1st trimester  2nd trimester  3rd trimester | 114  43  61 | 52.3  19.7  28 |

| Pregnant women experience food craving | Yes  No | 189  229 | 45.2  54.8 |
| --- | --- | --- | --- |
| Types of food craved | Meat  Cultural food  Egg  Injera  Bread | 118  29  24  12  6 | 62.4  15.3  12.7  6.3  3.2 |
| Reasons for food crave | Taste of food  Smell of food  Color of food  Other (like advice from others) | 84  65  20  20 | 44.4  34.4  10.6  10.6 |

Table 6: Factors associated with food taboos among pregnant women attending ANC follow-up in Deder town health facilities, Eastern Ethiopia, 2022.

| **Variables** | **Category** | **Food Taboo** | | **COR (95% CI** | **AOR (95% CI)** |
| --- | --- | --- | --- | --- | --- |
|  |  | **Yes**  ***No* (%)** | **No**  ***No* (%)** |  |  |
| Educational status | Unable to read and write  Primary school  Secondary school  College and above | 47(20.1%)  102(43.6%  59(25.2%)  26(11.1% | 21(11.4%)  73(39.7%)  54(29.3%)  36(19.6%) | 3.1(1.51, 6.37) *  1.94(1.07, 3.48)*  1.513(0.810, 2.83)  1:00 | 3.36(1.24, 9.16)**  1.636(0.726, 3.684)  0.99(0.433,2.279)  1:00 |
| Partner educational status | Unable to read and write  Primary school  Secondary school  College and above | 49(21%)  60(25.4%)  72(31%)  53(22.6%) | 38(20.7%)  17(9.2%)  72(39.1%)  57(31%) | 1.387(0.788, 2.440)  3.796(1.97, 7.3)*  1.07 (0.66, 1.77)  1:00 | 0.904(0.399, 2.046)  2.768(1.24, 6.2)**  0.817(0.41, 1.62)  1:00 |
| Residence | Rural  Urban | 144(61.5%)  90(38.5%) | 149(81%)  35(19%) | 2.661(1.692, 4.18)*  1:00 | 0.892(0.38, 2.09)  1:00 |
| ANC follow-up | Yes  No | 103(44%)  131(56%) | 109(59.2%)  75(40.8%) | 1:00  1.848(1.250, 2.734) | 1:00  2.044(1.27, 3.29)** |
| Food Aversion | Yes  No | 140(59.8%)  94(40.2%) | 78(42.4%)  106(57.6%) | 2.024(1.37, 2.99)  1:00 | 2.04(1.31, 3.20)**  1:00 |
| Food craving | Yes  No | 117(50%)  117(50%) | 72(39%)  112(61%) | 1.556(1.05, 2.3)*  1:00 | 1.484(0.872, 2.524)  1:00 |
| Additional meal practice | Yes  No | 102(43.6%)  132(56.4%) | 101(54.9%)  83(45.1%) | 1:00  1.575(1.068, 2.323) | 1:00  1.770(1.14, 2.76)** |
| Skipping meal | Yes  No | 69(29.5%)  165(70.5%) | 64(34.8%)  120(65.2%) | 1:00  0.784(0.519, 1.186) | 1:00  0.996(0.570, 1.743) |
| Coffee consumption | Yes  No | 131(56%)  103(44%) | 80(43.5%)  104(56.5%) | 1.653(1.12, 2.4)*  1:00 | 1.115(0.658, 1.89)  1:00 |
| Pica consumption | Yes  No | 61(26.1%)  173(73.9%) | 38(20.7%)  146(79.3%) | 1.355(0.854, 2.148)  1:00 | 1.293(0.730, 2.290)  1:00 |
| Knowledge status | Poor  Good | 150(64.1%)  84(35.9%) | 75(40.8%)  109(59.2%) | 2.595(1.74, 3.86)*  1:00 | 1.959(1.24, 3.09)**  1:00 |
| Attitudes status | Unfavorable  Favorable | 142(60.7%)  92(39.3%) | 84(45.7%)  100(54.3%) | 1.837(1.24, 2.72)*  1:00 | 1.909(1.22, 2.99)**  1:00 |

**COR:** Crude Odds Ratio; **AOR:** Adjusted Odds Ratio *: Significant at P-value <0.25; **: P-Value < **0.05** considered as statistically significant.
